# A systematic review of co-ethnic exposure and cardiometabolic outcomes in racial and ethnic minoritized populations

**DOI:** 10.3389/phrs.2026.1609690

**Published:** 2026-09-01

**Authors:** Marilyn Tseng, Ashley S. Watt, Anna K. Ostrander, Carolyn Y. Fang

**Affiliations:** 1 Department of Kinesiology and Public Health, California Polytechnic State University, San Luis Obispo, CA, United States; 2 Cancer Prevention and Control Program, Fox Chase Cancer Center, Philadelphia, PA, United States

**Keywords:** cardiometabolic health, ethnic density, minoritized populations, neighborhood, residential segregation

## Abstract

**Objectives:**

Urban residential characteristics, including racial and ethnic composition, may be associated with cardiometabolic health. We synthesized the literature on neighborhood co-ethnic exposure and cardiometabolic outcomes among racial and ethnic minoritized populations.

**Methods:**

We searched PubMed and Web of Science for studies published through October 2023 examining ethnic density or segregation and cardiometabolic conditions (adiposity, cardiovascular disease, and diabetes). Study quality was assessed using the U.S. National Institutes of Health’s Quality Assessment Tool.

**Results:**

Forty-five articles representing 34 unique studies were included. Over half relied on self-reported outcomes, and nine were longitudinal. The most common exposure measure was percentage of census tract residents sharing participant’s racial/ethnic classification. Over 40% of studies including Black and Hispanic/Latine populations reported an association of greater co-ethnic exposure with poorer cardiometabolic outcomes, compared with 7% of studies including Asian populations. Across all three groups, 25%–40% of studies reported inverse associations.

**Conclusion:**

Associations between neighborhood co-ethnic exposure and cardiometabolic outcomes vary both across and within racial/ethnic groups, reflecting interactions among structural (dis)advantage, community stressors, and neighborhood resources. Future research should integrate theoretical frameworks and sociohistorical context.

## Introduction

Cardiometabolic conditions are leading causes of morbidity and mortality and a substantial public health burden. Cardiometabolic health (CMH) may be affected by the urban residential environment [1, 2], including structural factors (e.g., economic opportunities, education quality), community stressors (e.g., crime, noise, pollution), and built environment characteristics (e.g., pharmacies, grocery stores, fast-food restaurants) [3].

Residential segregation, “the spatial and physical separation of where individuals live in residential space” [4], is a structural factor that gives rise to another defining characteristic of urban environments – ethnic composition. Although residential segregation has been linked to poorer health [5], previous reviews [6, 7] have also drawn attention to an “ethnic density” effect, in which “people in racial/ethnic minority groups are healthier when they live in areas with a higher concentration of people from their own racial/ethnic group” [7]. In this review, we use the term *co-ethnic exposure* to refer to residential exposure to others who share one’s racial or ethnic identity. We use this as an umbrella term encompassing ethnic density, residential segregation, and related spatial measures, while recognizing that these measures capture distinct dimensions of neighborhood ethnic composition.

**FIGURE 1:**
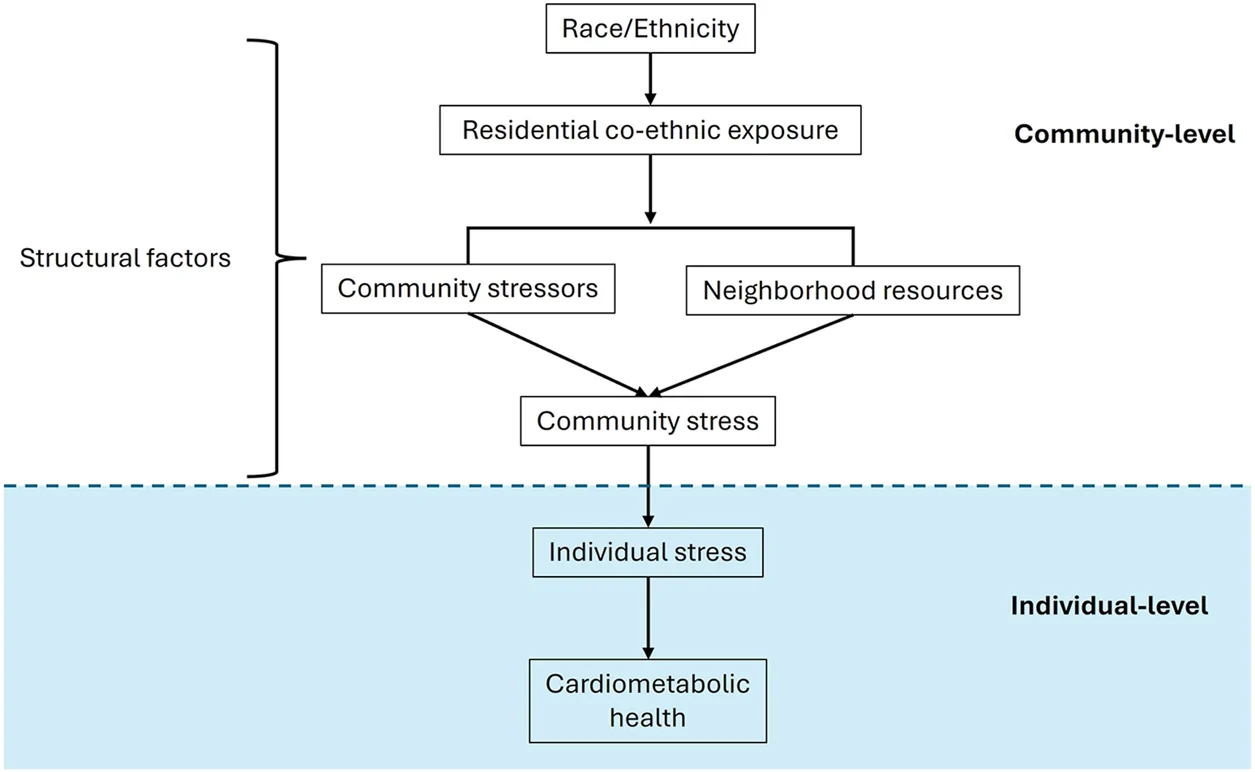
Conceptual framework linking residential co-ethnic exposure to cardiometabolic health, adapted from Gee and Payne-Sturges’s stress-exposure-disease model [3].

The stress-exposure-disease model proposed by Gee and Payne-Sturges [3] provides a basis for explaining heterogeneous associations between co-ethnic exposure and CMH (Figure 1). In this model, health outcomes in segregated environments result from the balance between community stressors and neighborhood resources, both of which are shaped by historically based structural factors affecting social, political, and economic conditions [3]. Thus, poorer CMH may result when community stressors arising from concentrated disadvantage and resource deprivation outweigh neighborhood resources [8], whether through behavioral coping strategies such as smoking or poor diet [4] or more directly through stress-related mechanisms in which glucocorticoids affect the regulation and functioning of multiple physiologic systems [3]. Conversely, protective associations may emerge if sharing an ethnic background and social identity with one’s neighbors contributes to feelings of connection and belonging, facilitates social support and informational networks, improves access to culturally sensitive health services, and fosters collective efficacy. These neighborhood resources may buffer or counterbalance structural disadvantage, thereby reducing stress and stress-related morbidities [8–10].

Previous reviews suggest worse physical health with co-ethnic exposure for Black populations and a possibly protective effect for Hispanic populations [7, 11], but findings for Hispanic populations are not clear [12] and may depend on factors such as country of origin [11]. Previous reviews also include few studies in other populations, and in the past decade since the last review was published, more studies have used novel measures for conceptualizing co-ethnic exposure. This review provides an updated synthesis of the literature on neighborhood co-ethnic exposure and cardiometabolic health-related conditions – in particular, adiposity, cardiovascular disease, and diabetes – in racial and ethnic minoritized populations.

## Methods

### Search strategy and eligibility criteria

We followed Preferred Reporting Items for Systematic Reviews and Meta-Analyses (PRISMA) guidelines for reporting the studies identified and included in this review [13]. Basic inclusion requirements delineated using the PECO (Population, Exposure, Comparator, Outcome) framework [14] are:

Population: We included studies reporting findings specific to racial and ethnic minoritized populations without restrictions on age or geographic area.

Exposure: Exposure measures included segregation or ethnic density measured at a geographical scale no larger than a U.S. county, metropolitan area, or equivalent unit. We acknowledge that residential segregation and ethnic density are distinct: Whereas residential segregation assumes a history of racial discrimination that forced minoritized people to live in certain areas, ethnic density is merely a measure of the percent of people of the same race or ethnicity in an area, which could have arisen from historical, discriminatory forces or from people’s own decisions to live around neighbors of similar ethnic background. We included both types of measures because our primary focus was whether the presence of others of the same minoritized race/ethnic group affects cardiometabolic health, regardless of the forces that led to higher co-ethnic exposure. Thus, we used the following search terms, connected with the Boolean operator “OR”: “ethnic enclave”, “ethnic density,” “residential segregation,” “racial segregation,” “neighborhood environment.”

Comparator: A comparable population (same racial or ethnic identity) with different levels of exposure.

Outcome: Cardiometabolic outcomes encompass many conditions. We focused on three – adiposity, cardiovascular disease, and diabetes – using the terms “weight,” “body mass index,” “obesity,” “cardiovascular disease,” or “diabetes.” We used broad terms with the expectation that they would capture the wide range of relevant outcomes. We excluded studies on management or treatment of these outcomes.

We conducted the literature search using the National Library of Medicine’s PubMed search engine, and the MEDLINE, Science Citation, and Social Sciences Citation (1996) indices of the Web of Science, including all articles published through October 2023 using the search terms given above (see Supplementary File). The electronic search was supplemented by manual review of reference lists from relevant articles and by use of “Cited by” or “Relevant articles” functions when available.

Articles were screened using these additional exclusion criteria: (1) not original research in a peer-reviewed journal; (2) ecologic study; (3) not in English.

### Study selection

**FIGURE 2:**
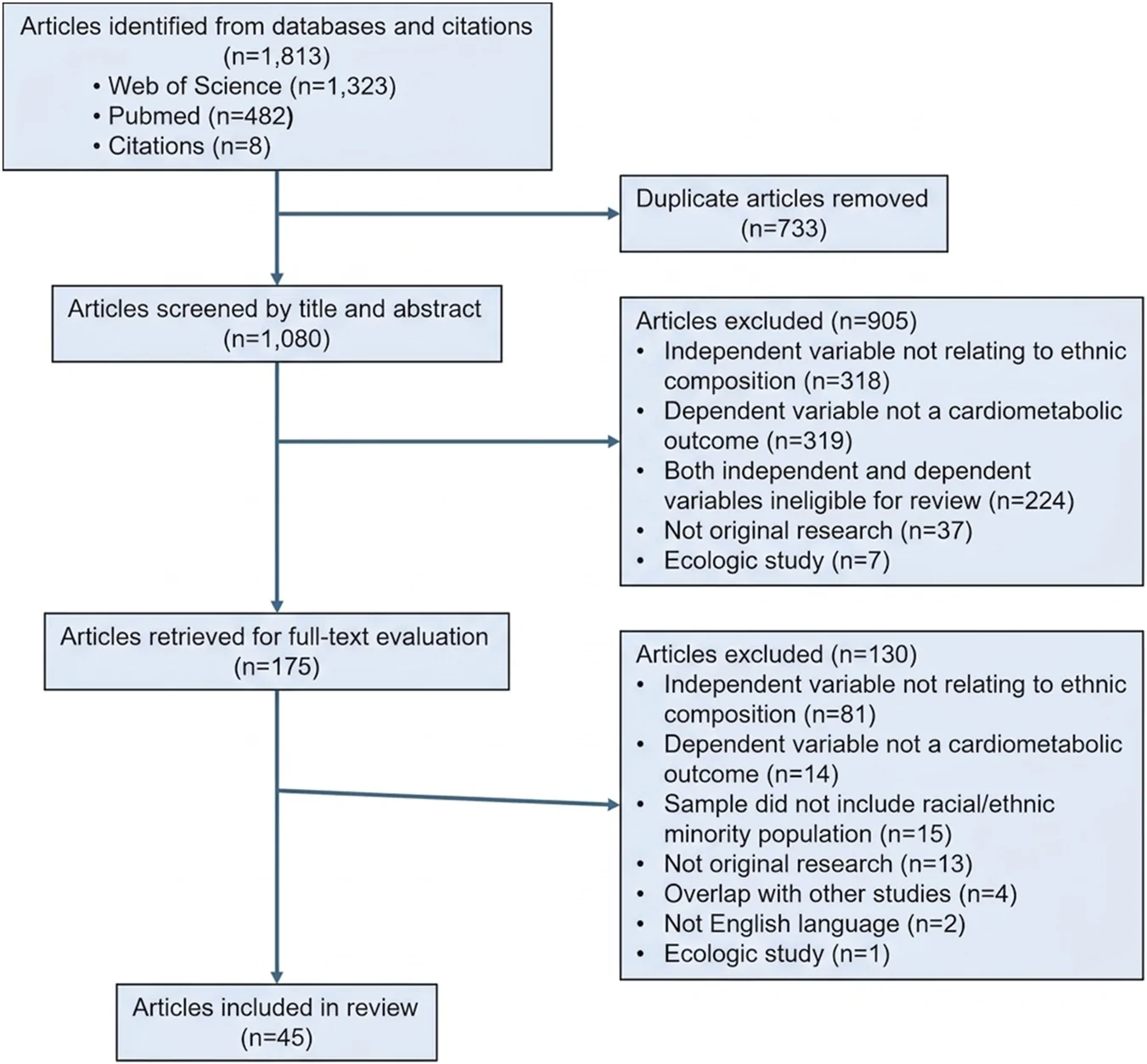
Flow diagram of articles identified, screened, and included in review.

Of 1,813 articles identified, 1,080 remained after removing duplicates (Figure 2). Four reviewers (A.O., M.S., A.T., A.S.W.) performed initial screening by title and abstract, with at least one person screening each record, resulting in removal of 905 articles. At least two reviewers evaluated the full text of each of the remaining 175 articles and made the selection decision by consensus, with disagreements resolved through consultation with the principal investigator (M.T.). Of these, 126 were removed, leaving 49. We further excluded four that examined subsets of samples in other included studies [15–18], leaving 45 articles. We then grouped together articles that examined different outcomes or race/ethnic groups but within the same source of data (e.g., NHANES 1999–2004; MESA; CARDIA), leaving a total of 34 unique studies.

### Data extraction and synthesis

Data were extracted from each study into tables that included: source of data; study design; description of sample (race/ethnic group, age, geographic location); measurement of co-ethnic exposure; area unit for measure of co-ethnic exposure; outcome variable(s); measurement of outcome variable(s) (self-reported, obtained from medical records, or measured); individual- and area-level covariates controlled for; and main findings. Findings were recorded as a positive association (higher occurrence with greater co-ethnic exposure), an inverse association (lower occurrence with greater co-ethnic exposure), or no association as reported by study authors based on their fully adjusted models, excluding adjustment for variables specified by the article’s authors as potential mediators. Findings were recorded as mixed if results depended on either level of exposure or an effect modifier. Each article’s data extraction was reviewed by at least two team members, and any problems or discrepancies were resolved by the principal investigator.

Our process for synthesizing study findings included tabulations, sorting articles according to study characteristics, then constructing a narrative synthesis of these results for Black, Hispanic/Latine, and Asian populations separately. To assess whether findings varied according to operationalization of co-ethnic exposure, we grouped measures into three categories: co-ethnic density (i.e., percent of same ethnicity), segregation measures, and local indicators of spatial association (LISA), then compared findings across these measurement categories. We used the 14-item Quality Assessment Tool for Observational Cohort and Cross-sectional Studies developed by the National Heart, Lung, and Blood Institute of the U.S. National Institutes of Health [19] to guide our assessment of study quality (Table 1). For each study we determined the percent of items that were adequately addressed. For longitudinal studies, this was calculated as the percent of all 14 items. Because three of the 14 items (7, 10, 13) were only relevant to longitudinal studies, for cross-sectional studies this was calculated as a percent of eleven items.

**TABLE 1:** Study quality assessment using the Quality Assessment Tool for Observational Cohort and Cross-sectional Studies^a^ [19].

Author	Dataset	Sampling^b^	Measurement of independent variable^c^	Measurement of dependent variable^d^	Confounding^e^	Longitudinal studies^f^	Score	Percent^g^
Longitudinal studies								
Cozier et al. [20]	Black Women’s Health Study, US, followed 1997 (ages 21–69) to 2009	+	++	–	++	++	10	71.4%
Jones et al. [21]	National Longitudinal Study of Adolescent to Adult Health (Add Health), US, followed 1995 (ages 12–18) to 2001–2002 (ages 18–26)	++	++	+	++	+	11	78.6%
Kershaw et al. [22] Do et al. [23] Mayne et al. [24] Gao et al. [25]	Multi-Ethnic Study of Atherosclerosis (MESA), six sites (New York, NY; Baltimore City and county, MD; Forsyth County, NC; St. Paul, MN; Chicago, IL; Los Angeles County, CA) followed 2000-2002 (age 45–84) to 2012 (2018 for Gao et al.)	++	++	++	++	+	11	78.6%
Kershaw et al. [26] Pool et al. [27] Mayne et al. [28] Reddy et al. [29]	Coronary Artery Risk Development in Young Adults (CARDIA), four field centers (Birmingham AL, Chicago IL, Minneapolis MN, or Oakland CA), followed from 1985 to 1986 (age 18–30)	++	++	+	++	+	11	78.6%
Mezuk et al. [30]	Nationwide registry data, Stockholm, Malmo, and Gothenburg, Sweden followed 2005 (age 30+) through 2010	++	+	+	++	–	8	57.1%
Moloney & South [31] Yang & South [32]	National Longitudinal Survey of Youth, 1979 Cohort (NLSY79) followed from 1979 (age 14–22)	++	++	–	++	++	11	78.6%
Pichardo et al. [33]	Hispanic Community Health Study/Study of Latinos (HCHS/SOL), Miami, FL, Chicago, IL, the Bronx, NY, and San Diego, CA, followed from 2008–11 (age 18–74) to 2014–17	++	++	+	++	–	9	64.3%
Schwartz et al. [34]	Panel Study of Income Dynamics Transition to Adulthood Study, US, followed 2005 (age 18–28) to 2017	++	++	–	++	++	11	78.6%
Wang et al. [35]	Panel Study of Income Dynamics (PSID) Child Development Supplement, US, followed 1997 (age 0–12) to 2014	++	++	–	++	+	10	71.4%
Cross-sectional studies								
Alvarez & Levy [36]	Established Populations for Epidemiologic Studies of the Elderly (EPESE), New Haven, CT, north central NC, 1982	+	++	–	–	​	4	36.4%
Astell-Burt et al. [37]	45 and Up Study, Australia 2006–2008	+	++	–	++	​	6	54.5%
Bravo et al. [38] Bravo et al. [39]	Electronic health records from Duke Medicine Data Warehouse, Durham County, NC 2007–2011	++	++	+	–	​	7	63.6%
Chang et al. [40]	Southeastern Pennsylvania Household Health Survey, Philadelphia, PA, 2002 and 2004	+	++	–	++	​	6	54.5%
Corral et al. [41] Corral et al. [42]	BRFSS, US 2000	+	++	–	++	​	6	54.5%
Do et al. [43]	National Health and Nutrition Examination Survey (NHANES), US 1988–94	++	++	+	++	​	8	72.7%
Do & frank [44]	National Health Interview Survey (NHIS), US (232 metropolitan areas with 100,000+ inhabitants, 5,000+ Hispanic) 2006–2013	++	++	–	++	​	7	63.6%
Eschbach et al. [45]	Hispanic Established Populations for Epidemiologic Studies of the Elderly (H-EPESE), Texas, California, Arizona, Colorado, New Mexico, 1993	++	++	–	++	​	7	63.6%
Gilbert et al. [46]	Convenience sample from outpatient primary care clinic serving medically underserved patients St. Louis, MO 2013–2014	+	+	+	–	​	5	45.5%
Janevic et al. [47]	Birth certificate and hospitalization data New York City 2001–2002	++	+	+	++	​	7	63.6%
Kershaw [48] Kershaw [49]	National Health and Nutrition Examination Survey (NHANES), US 1999–2006 (Kershaw et al. 2011 and Kershaw et al. 2013)	++	++	+	++	​	8	72.7%
Kershaw & albrecht [50]	BRFSS, US metro/micropolitan areas, 2003–2008	++	++	–	++	​	7	63.6%
Kirby et al. [51]	Medical Expenditure Panel Survey (MEPS), US 2002–2007	++	+	–	++	​	6	54.5%
Li et al. [52] Li et al. [53]	Southeastern Pennsylvania Household Health Survey, PA 2006 and 2008	+	+	–	++	​	5	45.5%
Lim et al. [54]	Community Health Surveys, New York City 2009–2012	+	+	–	–	​	4	36.4%
Mayne et al. [55]	Electronic health records from Northwestern Medicine Enterprise Data Warehouse for Prentice Women’s Hospital IL 2009–2013	++	++	+	++	​	8	72.7%
Mobley et al. [56]	Well-Integrated Screening and Evaluation for Women Across the Nation (WISEWOMAN), five US states states (Connecticut, Massachusetts, Nebraska, North Carolina, South Dakota) 2001–2002	–	+	+	++	​	4	36.4%
Nobari et al. [57]	Special Supplemental Nutrition Program for Women, Infants and Children data for Los Angeles County, CA, 2003–2009	+	++	+	++	​	7	63.6%
Park et al. [58]	Health survey, New York City 2000–2002	–	++	+	++	​	5	45.5%
Sutaria et al. [59]	Electronic health records from Clinical Effectiveness Group for 128 practices in three east London boroughs (Tower Hamlets, Newham, Hackney), UK 2014–2017	++	++	+	–	​	7	63.6%
Viruell-Fuentes et al. [60]	Chicago Community Adult Health Study (CCAHS) 2001–2003	++	++	–	++	​	7	63.6%
White et al. [61]	Community Health Surveys, New York City 2002 and 2005	++	+	–	++	​	6	54.5%
Williams et al. [62]	Consortium on Safe Labor, 19 hospitals in 15 Hospital Referral Regions in US, 2002–2008	++	+	+	++	​	7	63.6%
Wong et al. [63]	California Health Interview Survey (CHIS) 2011–2013	+	++	–	++	​	6	54.5%
Yu et al. [64]	BRFSS, US counties (n = 205) with African American population 5+% of total county population, 2012	+	++	–	++	​	6	54.5%

^a^ Criteria were based on the following items: 1. Was research question or objective clearly stated? 2. Was study population clearly specified and defined? 3. Was participation rate of eligible persons ≥50%? (Scored as “yes” if used existing health records.) 4. Were subjects selected or recruited from same or similar populations and time period? Were inclusion/exclusion criteria prespecified and applied uniformly? (Scored as “yes” if used all eligible individuals from existing records (e.g., medical or administrative database). 5. Was sample size justification, power description, or variance and effect estimates provided? 6. Was exposure measured prior to outcome? 7. Was timeframe sufficient to see association between exposure and outcome if it existed? (Scored for longitudinal studies only.) 8. Did study examine different levels of exposure (e.g., categories or as continuous variable)? 9. Was exposure measure clearly defined, valid, reliable, and implemented consistently across study participants? 10. Was exposure assessed more than once over time? (Scored for longitudinal studies only.) 11. Were outcome measures clearly defined, valid, reliable, and implemented consistently across study participants? (Scored as “no” if outcome was self-reported.) 12. Were outcome assessors blinded to exposure status? (Studies in which outcome was assessed prior to formulation of the co-ethnic exposure research question or in which outcome was self-reported were scored as “not applicable” (NA). Studies in which data (i.e., from medical records) were extracted by unknown investigators were marked as “not reported” (NR).) 13. Was loss to follow-up ≤20%? (Scored for longitudinal studies only.) 14. Were key potential confounding variables measured and adjusted for?

^b^ Based on four items (2, 3, 4, 5). 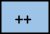 = 3–4 items addressed; 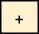 = 1–2 items addressed; 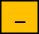
= 0 items addressed.

^c^ Based on two items (8, 9). 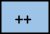 = 2 item addressed; 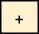 = 1 items addressed; 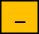 = 0 items addressed.

^d^ Based on two items (11, 12). 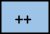 = 2 items addressed; 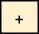 = 1 item addressed; 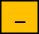 = 0 items addressed.

^e^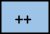 = item 14 addressed; 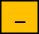 = item 14 not addressed.

^f^ Based on three items relevant to longitudinal studies only (7, 10, 13). 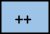 = 3 items addressed; 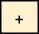 = 1–2 items addressed; 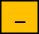 = 0 items addressed.

^g^ Calculated as percent of 14 items for longitudinal studies, 11 items for cross-sectional studies.

## Results

Of 34 unique studies (Table 2), nine included more than one CMH outcome. Body mass index (BMI) was the most commonly examined outcome (n = 24), followed by blood pressure (n = 11), and diabetes or gestational diabetes (n = 7). Two studies examined outcomes among children (<18 years) [35, 57], and three were conducted outside of the U.S. – Australia [37], Sweden [30], and the United Kingdom [59]. In terms of effect measures used, most studies reported odds ratios from logistic regression models. Others reported regression coefficients for continuous outcomes, and several reported hazard or rate ratios from longitudinal analyses.

**TABLE 2:** Studies of co-ethnic exposure and cardiometabolic health outcomes.

References	Dataset	Study sample	Co-ethnic exposure measure(s)	Area unit	Outcome(s)	Summary of findings
Longitudinal studies						
Cozier et al. 2014 [20]	Black Women’s Health Study, US, followed 1997 (ages 21–69) to 2009	Black: 12,810 Black women	% Black (quartiles)	Census block group	BMI	Incident rate ratio for highest vs. lowest quartile of neighborhood percent african American was 1.38 (95% CI: 1.12, 1.70, trend p = 0.01) in the subset of 3,550 women who remained in the same quartile over follow-up from 1997 to 2009
Jones et al. 2023 [21]	National Longitudinal Study of Adolescent to Adult Health (Add Health), US, followed 1995 (ages 12–18) to 2001–2002 (ages 18–26)	Hispanic/Latine: 2,445 (575 1st gen, 1,000 2nd gen, 870 3rd + gen) Asian: 1,061 (462 1st gen, 421 2nd gen, 178 3rd + gen)	Immigrant concentration scale (% foreign-born residents, % persons 5+ living in linguistically isolated household)	Census tract	BMI	No significant association in regression models
Kershaw et al. 2015 [22] Do et al. 2019 [23] Mayne et al. 2019 [24] Gao et al. 2022 [25]	Multi-Ethnic Study of Atherosclerosis (MESA), six sites (New York, NY; Baltimore City and county, MD; Forsyth County, NC; St. Paul, MN; Chicago, IL; Los Angeles County, CA) followed from 2000-2002 (age 45-84) to 2012 (2018 for Gao et al.)	Black, Hispanic/Latine, asian Kershaw: 1,595 Black, 1,289 Hispanic Do: 5,306 total: 1,628 Black, 1,310 Hispanic Mayne: 1,470 Black, 1,254 Hispanic Gao: 693 non‐Hispanic Black, 778 Hispanic, 466 Chinese	Getis-Ord local (G_i_*) statistic (<0, 0–1.96, >1.96)	Census tract and neighboring tracts vs. county	Kershaw: CVD Do: BMI Mayne: CMR Gao: Hypertension	Kershaw: Segregation was associated with higher hazard of CVD among Black participants (HR = 1.12, 95% CI 1.02–1.23, per standard deviation increase) but not Hispanic participants Do: Segregation was associated with BMI among Hispanic females in cross-sectional analyses (beta = 0.27, 95% CI 0.01–0.53, per 1.96 unit change) but not longitudinal analyses; it was associated with BMI among Black females in longitudinal analyses (beta = 0.24, 95% CI 0.03–0.46) Mayne: Segregation was associated with CMR at baseline among Black participants (beta = 0.17, 95% CI 0.02–0.32, for high vs. low segregation) Gao: Segregation was associated with hypertension in Black participants (HR = 1.24, 95% CI 1.01–1.54, for 1.96 vs.<=1.96), but not significantly among Hispanic or Chinese participants in fully adjusted models
Kershaw et al. 2017 [26] Pool et al. 2018 [27] Mayne et al. 2020 [28] Reddy et al. 2022 [29]	Coronary Artery Risk Development in Young Adults (CARDIA), four field centers (Birmingham AL, Chicago IL, Minneapolis MN, or Oakland CA), followed from 1985 to 1986 (age 18–30)	Black Kershaw: n = 2,280 followed 25 years Pool: n = 2,207 followed 25 years Mayne: n = 2,175 followed 30 years Reddy: n = 1,125 followed 15 years	Getis-Ord local (G_i_*) statistic (<0, 0–1.96, >1.96)	Census tract and neighboring tracts vs. MSA/county	Kershaw: BP Pool: BMI Mayne: Diabetes Reddy: coronary artery calcification	Kershaw: A 1-SD increase in segregation score was associated with a mean systolic blood pressure increase of 0.16 (95% CI, 0.06–0.26) mm Hg, and reductions in exposure to segregation among those who lived in high-segregation neighborhoods at baseline were associated with reductions in systolic blood pressure (for high- to low-segregation neighborhoods, −1.48, 95% CI -2.50 to −0.47) Pool: Among Black women, HR for obesity comparing high vs. low segregation neighborhood at prior exam 1.3 (95% CI 1.0–1.7); HR comparing cumulatively high vs. low exposure to segregation across time points 1.5 (95% CI 1.0–2.3). No association for men Mayne: No association between segregation and incident diabetes (HRs ∼1) Reddy: Living in low segregation neighborhoods in young adulthood associated with lower risk of developing CAC (rate ratio for low vs. high 0.52, 95% CI 0.28–0.98), attenuated to non-significance with adjustment for midlife risk factors
Mezuk et al. 2014 [30]	Nationwide Registry Data, Stockholm, Malmo, and Gothenburg, Sweden 2005 (age 30+) followed through 2010	Asian n∼12,400 (1.4% of 887,603 Native Swedes, Iraqi immigrants, and non-Iraqi immigrants)	Moran’s index used to identify 49 Iraqi immigrant enclaves	Swedish small area Market statistics	Diabetes	Enclave residence was not associated with odds of diabetes among 1st or 2nd generation Iraqi immigrants
Moloney & South 2015 [31] Yang & South 2018 [32]	National Longitudinal Survey of Youth, 1979 Cohort (NLSY79) followed from 1979 (age 14–22)	Black, Hispanic/Latine Moloney: 2,880 non-Hispanic Black, 1,878 Latina/o participants followed to 2004 Yang: 2,097 non-Hispanic black, 1,476 Latino participants followed to 2010	% co-ethnic density	Moloney: Metropolitan area or nonmetropolitan county Yang: Census tract	BMI	Moloney: At metropolitan area/county level, Black males are less likely to become obese when they live in areas with comparatively large representation of Black residents (OR = 0.053, p < 0.05, per standard deviation change in % co-ethnic density); no association for Black females or Latina/o males or females in adjusted, fixed effects models Yang: At the census tract level, Hispanic participants in neighborhoods with more Hispanic residents have higher BMIs (beta = 1.056 for cumulative exposure); no association among Black participants
Pichardo et al. 2023 [33]	Hispanic Community Health Study/Study of Latinos (HCHS/SOL), Miami, FL, Chicago, IL, the Bronx, NY, and San Diego, CA, followed from 2008-11 (age 18–74) to 2014–17	Hispanic/Latine: n = 6,710	Isolation index Gini coefficient (quintiles)	Census blocks aggregated over census tracts	Metabolic syndrome	Isolation index was associated with higher odds of metabolic syndrome (OR = 2.29, 95% CI 1.49–3.52 for fifth vs. first quintile), but gini coefficient was not
Schwartz et al. 2022 [34]	Panel Study of Income Dynamics (PSID) Transition to Adulthood Study, US, followed from 2005 (age 18–28) to 2017	Black: n = 1823	Getis-Ord local (gi*) statistic	Census tract and neighboring tracts vs. Core-based statistical area or county	BMI	Young Black adults who moved from low to high (OR = 0.41, 95% CI 0.25–0.65) or from high to low (OR = 0.64, 95% CI 0.42–0.98) segregation neighborhoods had lower odds of obesity than those who always lived in high segregation neighborhoods, but no difference in odds for those who always lived in low segregation neighborhoods
Wang et al. 2022 [35]	Panel Study of Income Dynamics (PSID) Child Development Supplement, US, followed from 1997 (age 0–12) to 2014	Black: n = 1,251	Getis-Ord local (G_i_*) statistic	Census tract and neighboring tracts vs. Core-based statistical area or county	BMI	No significant association between segregation and BMI in fixed effects models, but Black children who moved from low to high segregation neighborhoods were less likely than children who had always lived in low segregation neighborhoods to be overweight/obese (beta = -0.062, 95% CI -0.122 to −0.001)
Cross-sectional studies						
Alvarez & Levy 2012 [36]	Established Populations for Epidemiologic Studies of the Elderly (EPESE), New Haven, CT, north central NC, 1982	Black: n = 2,790 Age 65+	% Black (<25%, 25%–49%, 50+%)	County	CVD	Greater ethnic density predicted lower levels of cardiovascular disease in african Americans (OR 0.54, 95% CI 0.38–0.77 for 50+% vs. <25%)
Astell-Burt et al. 2013 [37]	45 and Up Study, Australia 2006–2008	Asian 2,230 Chinese born in China, 662 Chinese born in Australia 544 Lebanese born in Lebanon, 434 Lebanese born in Australia Age 45+	% co-ethnic density	Australian census collection district	BMI	Protective association between own-group ethnic density and continuous BMI for australian-born Chinese attenuated after adjusting for neighborhood affluence and geographical remoteness
Bravo et al. 2018 [38] Bravo et al. 2019 [39]	Electronic health records from Duke Medicine Data Warehouse, Durham County, NC 2007–2011	Black: n = 65,026 age 18+	Local indicator of spatial association (LISA)	Block	Bravo 2018: Diabetes Bravo 2019: Hypertension	Bravo 2018: Increased racial isolation was associated with diabetes (RR = 1.07, 95% CI 1.06–1.09, per 0.20 unit increase in racial isolation) Bravo 2019: Increased racial isolation was associated with hypertension (OR 1.06, 95% CI 1.03–1.10)
Chang et al. 2009 [40]	Southeastern Pennsylvania Household Health Survey, Philadelphia, PA, 2002 and 2004	Black: n∼2,500 age 18+	% Black (<20%, 20%–60%, >60%)	Census tract	BMI	For women but not men, living in a neighborhood with high vs. low racial isolation is associated with increased BMI (beta = 0.956, p < 0.01, comparing >60% vs. <20%); this was attenuated to non-significance with adjustment for neighborhood physical and social disorder, hypothesized mediators. No race-specific estimates provided, but interaction analyses indicated that association did not vary by race of individual resident
Corral et al. 2012 [41] Corral et al. 2014 [42]	BRFSS, US 2000	Black Corral 2012: n = 11,142 Hispanic/Latine: Corral 2014: n = 8,785 Age 18–99 years	Isolation index (<50, 50–59, 60+)	MSA	BMI	Corral 2012: In african Americans, odds of overweight/obesity were significantly higher in high vs. low-segregated MSAs (OR = 1.175, 95% CI 1.056–1.307, for isolation index 60+ vs. < 50) Corral 2014: In Hispanic adults, odds of obesity were significantly higher in high vs. low-segregated MSAs (OR = 1.264, 95% CI 1.000–1.598)
Do et al. 2007 [43]	National Health and Nutrition Examination Survey (NHANES), US 1988–94	Black: n = 4,042 Hispanic/Latine: n = 3,973 Mexican Americans Age 20+	% co-ethnic density	Census tract	BMI	Higher proportion of Black residents in a neighborhood was associated with higher BMI for Black males (beta = 0.150, p < 0.01, per 10 percentage point change in % Black). Proportion Hispanic was not associated with BMI for Hispanic participants
Do & frank 2020 [44]	National Health Interview Survey (NHIS), US (232 metropolitan areas with 100,000+ inhabitants, 5,000+ Hispanic) 2006–2013	Hispanic/Latine: n∼26,700 age 25+	Isolation index Dissimilarity index	Census tracts aggregated over metropolitan area	BMI	Segregation is protective for Hispanic immigrants in low poverty neighborhoods (OR = 0.88, 95% CI 0.78–0.99, per 10 percentage point change in dissimilarity index), but increases obesity for US-born hispanics in high poverty neighborhoods (OR 1.10, 95% CI 1.01–1.20)
Eschbach et al. 2004 [45]	Hispanic Established Populations for Epidemiologic Studies of the Elderly (H-EPESE), Texas, California, Arizona, Colorado, New Mexico, 1993	Hispanic/Latine n = 2,669 Mexican Americans age 65+	% Mexican American	Census tract	Diabetes Stroke Heart attack Hypertension	Increasing percentage of Mexican Americans in neighborhoods was associated with decreasing prevalence of stroke (OR = 0.33, 95% CI 0.16–0.71, for 100% vs. 0% Mexican American), but not significantly associated with heart attack, hypertension, or diabetes
Gilbert et al. 2015 [46]	Convenience Sample from Outpatient Primary Care Clinic Serving Medically Underserved Patients, St. Louis, MO 2013–2014	Black: 111 non-Hispanic Black men age >18	% Black, self-reported (‘mostly blacks, some blacks, mostly whites, about half whites’), childhood and current neighborhoods	Self-defined neighborhood	BMI Hypertension	Those who grew up in mostly Black neighborhoods were more likely to be diagnosed with hypertension than those who did not (OR = 3.37, 95% CI 1.12–10.17, for mostly Black vs. other neighborhood types)
Janevic et al. 2014 [47]	Birth Certificate and Hospitalization Data, New York City 2001–2002	Immigrant women Black 16,339 non-Hispanic Caribbean 2,951 sub-Saharan african Hispanic/Latine 20,680 Central/South American 16,423 Dominican 13,370 Mexican 4,432 Puerto rican Asian 10,603 Chinese 9,920 South Central asian	% co-ethnic density (95th percentile cutpoint, ranging from 2.0% for non-Hispanic Caribbean to 36.3% for Puerto rican women)	Census tract	Gestational diabetes	Living in a residential ethnic enclave was associated with increased odds of gestational diabetes among South Central asian (OR = 1.24, 95% CI 1.07–1.44) and Mexican women (OR = 1.28, 95% CI 1.08–1.51)
Kershaw 2011 [48] Kershaw 2013 [49]	National Health and Nutrition Examination Survey (NHANES), (NHANES), US 1999–2006	Black, Hispanic/Latine Kershaw 2011: n = 2,382 Black adults age 25+ Kershaw 2013: n = 2,660 Black and n = 2,611 Mexican American adults age 25+	Isolation index	Census tracts aggregated over MSA	Kershaw 2011: Hypertension Kershaw 2013: BMI	Kershaw 2011: Increased segregation was associated with higher odds of hypertension (OR = 1.18, 95% CI 1.00–1.39, per standard deviation increase) among Black participants Kershaw 2013: Increased segregation was associated with higher prevalence of obesity (prevalence ratio = 1.30, 95% CI 1.02–1.67, for isolation index >0.6 vs.<=0.30) among Black women. It was associated with lower prevalence of obesity among Mexican American women (prevalence ratio = 0.56, 95% CI 0.33–0.93, for isolation index >0.6 vs.<=0.30)
Kershaw & Albrecht 2014 [50]	BRFSS, US metro/micropolitan areas, 2003–2008	Black: n = 2,263 Hispanic Black Hispanic/Latine: n = 57,883 Hispanic white or other race age 25+	Isolation index	Census tracts aggregated over metro- or micro-politan statistical areas (MMSA)	BMI	No association between segregation and BMI in men. Among women, higher segregation was associated with higher mean BMI for Hispanic White women (beta = 0.17, p = 0.11) but with lower BMI for Hispanic Black women (beta = -0.54, p = 0.12, interaction p = 0.03)
Kirby et al. 2012 [51]	Medical Expenditure Panel Survey (MEPS), US 2002–2007	Black: n = 18,883 Hispanic/Latine: n = 28,500 Asian: n = 5,475	% co-ethnic density (25+%)	Census block-group	BMI	For Hispanic participants, living in communities of 25+% vs. <25% of Hispanic residents was associated with higher BMI and higher odds of obesity (OR = 1.21, p < 0.05). Co-ethnic density was not associated with BMI in non-Hispanic Black or asian participants
Li et al. 2014 [52] Li et al. 2017 [53]	Southeastern Pennsylvania Household Health Survey, PA 2006 and 2008	Black Li 2014: n = 4,290 Hispanic/Latine Li 2017: n = 1,563	% co-ethnic density (25%) % foreign-born tertiles (li 2017 only)	Census tract	Li 2014: BMI Li 2017: High blood pressure High cholesterol	Li 2014: No association (ORs ∼1) between co-ethnic density and obesity was found for Black participants Li 2017: High Latino density was associated with increased likelihood of having high cholesterol level (OR = 1.47, 95% CI 1.02–2.14, for 25+% vs. <25%). High immigrant concentration was associated with significantly decreased likelihood of high blood pressure and high cholesterol, but propensity score matching analysis suggested possibility of selection bias
Lim et al. 2017 [54]	Community Health Surveys, New York City 2009–2012	Asian: n = 2,863	Isolation index and dissimilarity index (scores >0.35) used to identify five community districts as asian enclaves (Chinatown, fresh Meadows, flushing, Sunset Park, Elmhurst)	Census tracts aggregated over community districts	Diabetes Hypertension	Adjusted prevalence estimates of hypertension and diabetes were not associated with living in an ethnic enclave
Mayne et al. 2018 [55]	Electronic Health Records from Northwestern Medicine Enterprise Data Warehouse for Prentice Women’s Hospital, Chicago, IL 2009–2013	Black: 4,748 non-Hispanic Black women who gave birth to one child in Prentice Women’s Hospital in Chicago, IL	Getis-Ord local (G_i_*) statistic (<0, 0–1.96, >1.96)	Census tract and neighboring tracts vs. Chicago MSA	Hypertension in pregnancy	Residential segregation was associated with lower odds of hypertensive disorder of pregnancy at low levels of poverty (e.g., OR ∼0.5 at 0% neighborhood poverty, p < 0.05) but with higher odds at higher levels of poverty (e.g., OR ∼2.0 at 25% neighborhood poverty, p < 0.05, interaction p = 0.002)
Mobley et al. 2006 [56]	Well-Integrated Screening and Evaluation for Women Across the Nation (WISEWOMAN), five US states (Connecticut, Massachusetts, Nebraska, North Carolina, South Dakota) 2001–2002	Black: n∼350 non-Hispanic Black Hispanic/Latine: n∼485 Asian: n∼27 American Indian: n∼108 uninsured, low income women age 20–64	Isolation index	Zip code area	BMI CHD (algorithm including gender, age, total and high-density lipoprotein cholesterol, systolic blood pressure, smoking status, and diabetes status)	Racial segregation was not associated with BMI for women of any race, but there were significant negative associations between racial/ethnic segregation and CHD risk among black (beta = -0.52), Hispanic (beta = -0.61), and asian (beta = -2.44) women and a significant positive association among American Indian women (beta = 1.91)
Nobari et al. 2013 [57]	Special Supplemental Nutrition Program for Women, Infants and Children data for Los Angeles County, CA, 2003–2009	Hispanic/Latine: n = 116,362 children of Spanish-speaking mothers Asian: n = 1,299 children of Chinese-speaking mothers Age 2–5 years	% Residents preferring to speak Spanish or Chinese	Census tract	BMI	Among asian children, percent of neighborhood residents who spoke Chinese was negatively associated with BMI (beta = -0.004, p = 0.056). Among Hispanic children, the association was positive at lower levels of percent of Spanish-speaking residents and negative at higher levels
Park et al. 2008 [58]	Health Survey, New York City 2000–2002	Black: n = 638 Black Caribbean Hispanic/Latine: n = 2,616 Asian: n = 1,530	% co-ethnic density % foreign-born % Linguistically isolated	Census block groups within ½-mile radial buffer around residential address	BMI	Increasing linguistic isolation was inversely associated with BMI among Hispanic participants (beta = -2.97, p = 0.03, per 1-unit change in household linguistic isolation). No associations for co-ethnic density or immigrant composition for any groups
Sutaria et al. 2019 [59]	Electronic health records from clinical Effectiveness group for 128 practices in three east London boroughs (Tower Hamlets, Newham, Hackney), UK 2014–2017	Black: n = 30,761 Black africans Asian Bangladeshi n = 51,575 Indian n = 29,250 Pakistani n = 16,884 Age 18+	% co-ethnic density	Middle Super Output area	BMI	An increase in black african ethnic density was associated with increased odds of obesity (OR = 1.15, 95% CI 1.07–1.24 for males, OR = 1.18, 95% CI 1.08–1.30, per 10% increase in co-ethnic density). An increase in Indian ethnic density was associated with a decrease in odds of obesity among Indian women (OR = 0.93, 95% CI 0.88–0.99). Associations were limited to individuals age >35
Viruell-Fuentes et al. 2012 [60]	Chicago Community adult health study (CCAHS) 2001–2003	Hispanic/Latine: 804 Latinos	Racial/ethnic/immigrant composition from principal factor analysis (% Hispanic, % foreign-born)	Census tract	Hypertension	Greater concentrations of Latino residents and immigrants in the neighborhood were associated with lower odds of having hypertension (OR = 0.60, p = 0.03)
White et al. 2011 [61]	Community health Surveys, New York City 2002 and 2005	Black: n = 4,499 age 18+	Wong’s index	Census tracts aggregated over United Hospital fund neighborhood designations	Hypertension	Foreign-born Black participants age 65+ residing in highly segregated areas (Wong’s index at or above median of 0.55) were less likely to report hypertension than their counterparts in less segregated areas (prevalence ratio = 0.54, 95% CI 0.40–0.72). No association between segregation and hypertension in US-born Black or younger foreign-born Black participants
Williams et al. 2021 [62]	Consortium on Safe Labor, 19 hospitals in 15 Hospital referral regions in US, 2002–2008	Asian/Pacific Islander: n = 8,350 women	Ethnic enclave defined based on being in upper tertile for % API, API-White dissimilarity index, and API isolation index	Hospital referral region	Gestational diabetes	Residence in an ethnic enclave was associated with lower odds of gestational diabetes compared to residence in a non-enclave (ORs varying depending on volatile organic compound stratum)
Wong et al. 2018 [63]	California health Interview survey (CHIS) 2011–2013	Black: n = 2,943 Hispanic/Latine: n = 13,466 Asian: n = 5,499 Age 18+	% co-ethnic density	Census tract	BMI	High proportion of asian residents in the neighborhood was associated with lower odds of obesity (OR = 0.87 per 10% change, 95% CI 0.79–0.96). No association for Black or Hispanic participants
Yu et al. 2018 [64]	BRFSS, US Counties (n = 205) with African American Population 5+% of total County Population, 2012	Black: n = 21,865 Hispanic/Latine: n = 18,027 Asian: n = 4,505 Age 18+	Segregation isolation index Dissimilarity index Concentration index	Census tracts aggregated over county	BMI	African American and Hispanic participants in counties with high levels of isolation, dissimilarity, and concentration were more likely to be obese (ORs per unit change ranging from 1.257 to 1.808 depending on segregation measure and race/ethnic group, all p < 0.05); no association for asian participants

### Study quality issues

In our summary of assessment of study quality (Table 1), longitudinal and cross-sectional studies are shown separately. For purposes of presentation, we grouped together four items (items 2–5) relating to sampling, two items (items 8–9) relating to independent variable measurement, and two items (items 11–12) relating to measurement of the outcome. Scores for longitudinal studies (mean 10.2, or 73% of 14) were generally higher than for cross-sectional studies (mean 6.2, or 56% of 11). Study quality scores did not differ appreciably across study populations (not shown).

All studies clearly stated their research questions (not shown), most (29 of 34, or 85%) adjusted for both individual-level social position and neighborhood-level socioeconomic disadvantage, and study populations were mostly clearly defined, with consistent recruitment procedures and application of inclusion and exclusion criteria. Eleven of 25 cross-sectional studies did not report participation rates (n = 6) or reported participation rates <50% (n = 5). Only one study directly addressed sample size and power [33]. Sample sizes varied from ∼30 to >116,000 participants, with median sample sizes across studies ranging from 3,000 to 4,000 across racial/ethnic groups. Twelve (35%) of the 34 studies used existing data, such as the Behavioral Risk Factor Surveillance System (BRFSS) or routinely collected health data, containing relatively large (n > 10,000) samples for at least one subpopulation (Black, Hispanic/Latine, Asian).

Measures of the independent variable were deemed to be valid and reliable overall: Most were based on data for an administratively defined geographic unit (e.g., census tract), and most studies also examined levels of exposure in the form of continuous or categorical variables. With respect to the dependent variables, however, over half (53%) of the studies (n = 18) relied on self-reported measures. Only one [22] explicitly addressed blinding in assessing the outcome, although in many studies it is likely that the outcome was assessed before the research question on co-ethnic exposure was developed.

Finally, only nine (26%) of the 34 studies were longitudinal. For seven (78%) of these, length of follow-up was deemed adequate given the outcome and the average cohort age at baseline, with a median follow-up of 12 years (range 4–25 years). In six (67%) of the studies, exposure was assessed more than once, with a median of one assessment every 3 years. Only three longitudinal studies reported an attrition rate of <20%.

### Measures of co-ethnic exposure

**FIGURE 3:**
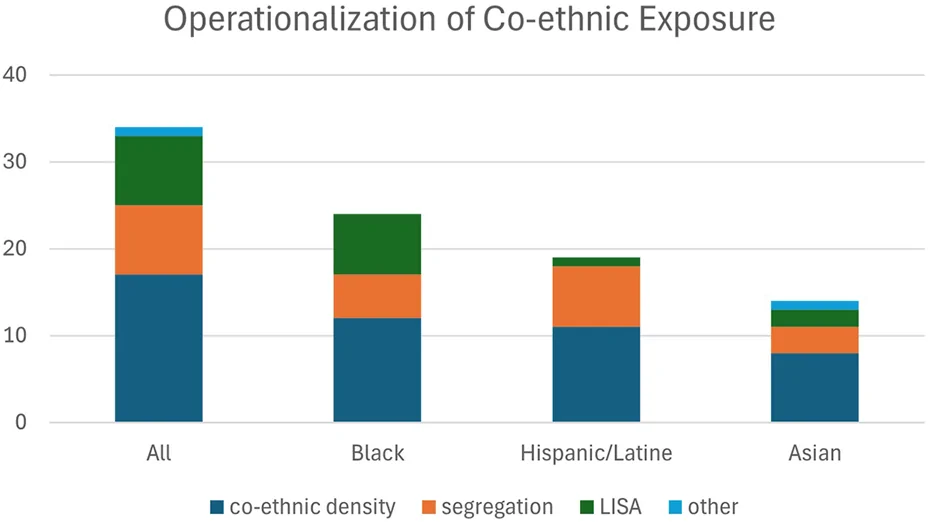
Distribution of how co-ethnic exposure was operationalized overall and by race/ethnic group: as measure of co-ethnic density, segregation, local indicator of spatial autocorrelation (LISA), or other (combination of co-ethnic density and segregation).

The most common approach, used in half of the 34 studies (Figure 3), involved calculating the percentage of residents in a census tract who were of the same racial/ethnic classification as the participant. Percent ethnic density was analyzed as a continuous variable in some studies (e.g., [37, 43, 59, 63]), as quartiles [20], or as dichotomous variables using cutpoints that ranged from <5% to 65%. Variations included using language preference instead of racial/ethnic classification [57]; combining information on ethnic density with immigrant concentration and/or household linguistic isolation [21, 60], or eliciting participant perceptions on the racial composition of their neighborhood [46].

Eight studies measured level of segregation for a larger geographic area (i.e., metropolitan area) aggregated over smaller areas (i.e., census tracts). The main dimension of segregation in these studies was exposure, or the level of potential contact between groups, quantified using the isolation index. Lim et al. supplemented their use of the isolation and dissimilarity indexes with an examination of their spatial overlap with percent density and language use to identify five specific areas in New York City as Asian enclaves [54].

Another eight studies used local indicators of spatial autocorrelation (LISA), which integrate a spatial dimension in describing degree of segregation of populations in small areas (i.e., census tracts) relative to the average for the larger area (e.g., metropolitan area or county). The Getis-Ord statistic was the most frequently used LISA. One study [62] used a combination of percent density and segregation measures to identify areas as ethnic enclaves.

More of the studies in Black populations used a LISA (29% vs. 5% in Hispanic/Latine and 15% in Asian populations), while more of the studies in Hispanic/Latine populations used a measure of segregation (37% vs. 21% in Black and Asian populations) (Figure 3).

### Studies by race/ethnic group

Given differences in their historical and socioeconomic contexts, findings are presented separately for three broad race/ethnic groups: Black, Hispanic/Latine, and Asian populations. The only study that included an American Indian or Alaska Native (AI/AN) sample [56], a longitudinal study of clinically measured cardiovascular risk, found that among AI/AN participants, segregation was associated with higher BMI but was not associated with diabetes.

#### Black populations

**FIGURE 4:**
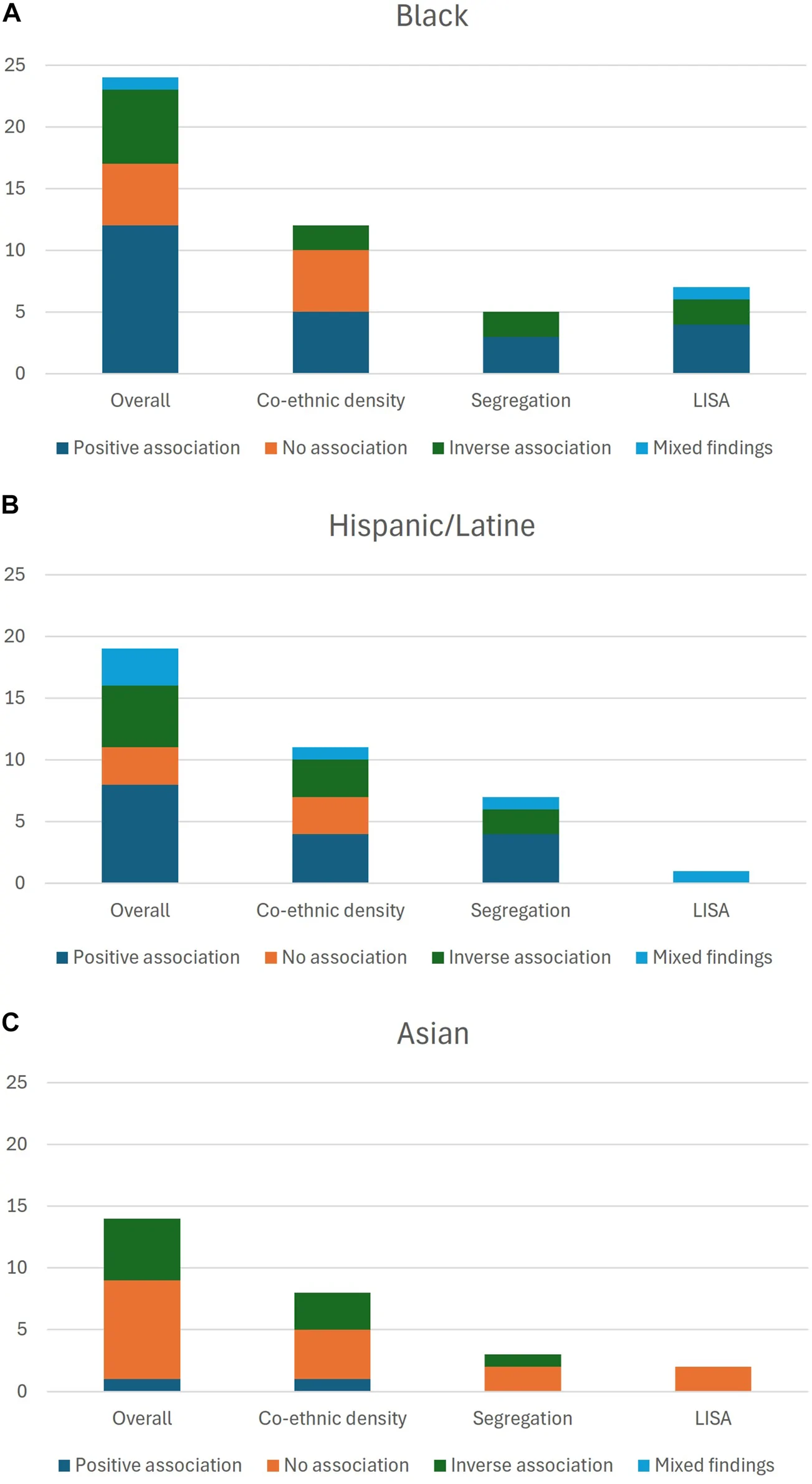
Distribution of associations between co-ethnic exposure and cardiometabolic outcomes (positive, inverse, mixed, or no association) for **(A)** Black, **(B)** Hispanic, and **(C)** Asian populations, overall and by operationalization of co-ethnic exposure [co-ethnic density, segregation, or local indicator of spatial autocorrelation (LISA)]. Figure for Asian populations excludes one study that used a combination of co-ethnic density and segregation [62], which found an inverse association.

Twelve (50%) of 24 studies in Black populations found a positive association, six (25%) an inverse association, and five (21%) no association (Figure 4A). In one cross-sectional study with mixed findings [55], local segregation measured using the G* statistic was associated with increased hypertension in pregnancy in neighborhoods of higher poverty but inversely associated in low-poverty neighborhoods.

Only one [49] of the five U.S. studies that included Black immigrant populations [47, 49, 50, 58, 61] saw higher occurrence of CMH outcomes with greater segregation, while two [50, 61] found an inverse association. In a study in NYC, the inverse association between a LISA and self-reported hypertension was observed *only* among older, foreign-born and not among US-born Black residents [61].

Cross-sectional studies were less likely than longitudinal studies to find a positive association [eight (44%) of 18 vs. four (67%) of six] and more likely to show no association [four (22%) of 18 vs. 0 of six]. Over half of the cross-sectional studies used measures of percent density, while more longitudinal studies used a LISA. Studies using percent density as their independent variable were more likely to show no association than studies relying on segregation or LISA measures (Figure 4A).

Most studies controlled for area-level poverty or some other indicator of socioeconomic disadvantage, and all controlled for individual-level characteristics. Four articles did not control for any area-level characteristics. Excluding these from our synthesis slightly decreased the proportion of studies reporting a positive association (nine (45%) of 20).

#### Hispanic/Latine populations

Of 19 studies that included Hispanic/Latine populations, eight (42%) reported a positive association, five (26%) an inverse association, and three (16%) no association (Figure 4B). Excluding the one study that did not adjust for area-level poverty did not materially change these percentages.

Results were mixed in three studies. In a study of children in Los Angeles County, Nobari et al. [57] described an inverted U-shaped association between proportion of Spanish-speaking residents and children’s BMI. In MESA 2000–2012 data, Do et al. [23] found a positive, cross-sectional association between the G* statistic and BMI among women but a suggestive inverse association for both women and men in longitudinal analyses. In NHIS 2006–2013 data [44], MSA-level segregation was positively associated with obesity for US-born Hispanic participants in high-poverty neighborhoods but inversely associated among Hispanic immigrants in low-poverty neighborhoods.

Five other studies presented results distinguishing Hispanic/Latine participants by nativity (US or foreign-born) [21, 33, 47, 49, 58]. Four saw no evidence of effect modification by nativity [21, 33, 49, 58], with two showing an inverse association, one a positive association, and one no association. The remaining study included only immigrants [47] and observed increased risk for gestational diabetes for mothers of Mexican but not Dominican, Puerto Rican, and Central or South American heritage. In three other studies that referred specifically to Mexican Americans (regardless of nativity) [45, 47, 49, 65], two observed inverse associations with stroke [45] and hypertension [49], and one observed no association with BMI [65].

When examined by how the independent variable was operationalized, the seven studies that used a segregation measure were more likely to show associations indicating increased occurrence of CMH outcomes (57%). The only studies that reported no association were 27% of the studies that relied on measures of percent density (Figure 4B).

#### Asian populations

Of 14 articles including Asian populations, three focused on Chinese [25, 37, 57], one included Chinese and South Asian women [47], one included Indian, Pakistani, and Bangladeshi women in London [59], and one focused on Iraqi immigrants in Sweden [30]. The other seven examined Asian samples more generally, with one also including Pacific Islanders [62].

Eight (57%) studies showed no association, five (36%) found an inverse association, and only one (7%) showed a positive association (Figure 4C); these proportions did not change meaningfully when we excluded two articles that did not adjust for area-level characteristics. All three longitudinal studies showed no association. The one positive association was observed between ethnic density and gestational diabetes among South Central Asian immigrant women in New York City; the same study found no association for Chinese immigrant women [47]. There were too few studies to discern any patterns with respect to study population, health outcome, measurement of the independent variable (Figure 4C), or whether the outcome was self-reported.

## Discussion

A major finding is that the association between residential co-ethnic exposure and CMH differs depending on the race/ethnic group. Whereas over 40% of studies in Black and Hispanic/Latine populations observed greater occurrence of CMH outcomes with higher co-ethnic exposure, only one of 14 studies including Asians did. Nevertheless, a meaningful proportion of studies across all groups – 25%–40% – found lower occurrence of CMH outcomes with higher co-ethnic exposure.

A framework for interpreting these heterogeneous findings comes from Gee and Payne-Sturges’ stress-exposure–disease model [3], in which community stressors and neighborhood resources interact within a broader structural context. Applied here, co-ethnic exposure may be understood not as inherently protective or harmful. Rather, its association with CMH may depend on the interaction between community-wide stressors such as concentrated disadvantage, disinvestment, and exclusion from opportunities and resources, and the resources available within a neighborhood such as shared identity, cultural continuity, social capital, mutual support, and collective efficacy. Because both stressors and resources may be present within the same neighborhood, the health implications of co-ethnic exposure likely depend on their relative influence and on the historical and social conditions that produced that neighborhood. This perspective helps explain why higher co-ethnic exposure was associated with both greater and lower cardiometabolic risk across studies.

Kramer [4] has asserted the importance of considering the historical processes that “produced – and may continue to reproduce” current residential patterns [4] with their concentration of social disadvantage, disenfranchisement, and limited access to wider networks of health-promoting opportunities and resources [8, 40, 43]. These historical processes may also contribute to the development of adaptive social norms and health behaviors [66, 67] with potentially deleterious health consequences [68, 69]. Further, the stress engendered in such segregated environments has measurable physiological effects [70, 71] with established mechanisms [70, 72], including trans-generational effects. A growing body of work [73–76] supports the possibility that among minoritized individuals with longer generational ties in the U.S., chronic and long-term exposure to stress and socioeconomic disadvantage across generations can accelerate aging, leading to poorer health outcomes. Taken together, these mechanisms may explain the findings in studies of Black populations showing poorer CMH with greater segregation.

In immigrant neighborhoods without a history of concentrated disadvantage and disenfranchisement, social resources and a sense of shared identity and belonging may have salutary effects for mental as well as physical health [6, 7]. Studies have generally found beneficial associations between social capital and health across U.S. communities [77–81], as well as in Asia [82–84], Europe [85], and South America [86, 87].

Variations in historical context across subpopulations within the same broad ethnic category might contribute to both the positive and inverse associations observed in studies among Black and Hispanic/Latine populations. ‘Hispanic’ communities, for example, include neighborhoods of different cultural heritage and national origin [88], ranging from communities in the Southwest that pre-date non-Hispanic White settlement to more recent immigrant destinations [89]. These different histories likely produced different present-day socioeconomic circumstances and resources, with different implications for health. Thus, both concentration of disadvantage and concentration of social capital may be true for Black and Hispanic/Latine populations depending on their histories [90].

### Strengths and limitations

This review incorporates several large and longitudinal studies, with a wide range of methods to operationalize the concept of co-ethnic exposure. Thus, we were able to discern different patterns of findings across Black, Hispanic/Latine, and Asian populations. In fact, a notable pattern emerged across measurement approaches: Among Black and Hispanic/Latine populations, studies using segregation-based measures more frequently reported adverse cardiometabolic associations than studies relying on measures of ethnic density, which more often yielded null findings. This suggests that different measures of co-ethnic exposure capture different dimensions of neighborhood context.

As a limitation, we did not include an exhaustive list of condition-specific search terms and therefore may not have identified some relevant studies. We attempted to minimize this possibility through review of reference lists and use of ‘Cited by’ and related-article functions, and we were able to capture a wide range of outcomes, including high blood pressure and other cardiovascular risk indicators. Another limitation is the challenge of synthesizing findings given heterogeneity in how co-ethnic exposure was operationalized. Although some patterns were discernible even with such heterogeneity, factors contributing to differences in findings across measures of co-ethnic exposure merit further investigation. For example, investigators’ selection of a particular measure may be influenced by their understanding of the processes that led to co-ethnic exposure, resulting in more frequent selection of segregation measures in studies including Black populations. Publication bias is another possible limitation if studies reporting statistically significant or theoretically expected associations were more likely to be published, although a substantial proportion of studies included in our review reported null or mixed findings, suggesting that publication of non-significant results does occur in this literature.

Other limitations relate to the evidence base. Of particular note is the dearth of studies in Asian populations, despite the fact that Asians comprise ∼30% of immigrants to the U.S. and were the largest source of immigrants to the US through the 2010s [91]. We identified only one study that included Pacific Island participants and another that included AI/AN participants. This critical evidence gap limits our understanding of how co-ethnic exposure affects CMH in these populations and reflects a broader gap in research on groups that are frequently aggregated or insufficiently represented in studies of neighborhood effects and health. As a result, it remains unclear whether the patterns observed among Black and Hispanic/Latine populations generalize to other racialized and Indigenous populations. Moreover, because our findings suggest that the health implications of co-ethnic exposure depend on historical and social context, the lack of studies in Pacific Island and Indigenous populations represents a particularly important gap in knowledge.

Also with respect to the evidence base, over half of the studies relied on a self-reported outcome, although we observed no clear differences in findings based on this study characteristic. That most studies were cross-sectional is another limitation discussed in more detail below.

Finally, although not a limitation *per se*, our findings should be interpreted in light of the scope of this review. For example, we focused on CMH, but other work provides evidence of associations with cancer [92] and mental health outcomes [93]. This review is also limited to national contexts in which White populations are the dominant racial group. The extent to which these findings relate to settings where non-White groups are dominant has yet to be explored. Finally, the review was limited to the experience of racial and ethnic minoritized populations, but segregation and co-ethnic exposure may also affect the health of non-minoritized populations in complex ways relating to availability of resources and expectations set up by social norms [94].

### Considerations for future research

More studies among Asian, Pacific Island and AI/AN populations are warranted, especially given their high risk for cardiometabolic disease [95, 96]. Another basic recommendation to strengthen the evidence base is to capture objective rather than self-reported outcome measures – for example wearable devices to capture measures of heart rate, cardiac electrical activity, and glucose levels [97]. Other considerations are discussed in more detail below.

#### Operationalization of co-ethnic exposure

Ethnic density was the most common measure, but different levels of ‘density’ may carry different meaning in different contexts. For example, the cutpoints used to represent ‘higher’ ethnic density ranged from <5% to 65% across studies, and the meaning of living in a neighborhood with an ethnic density of 25% varies across regions.

The selection of measure also depends on the spatial scale at which mechanistic factors are thought to operate. For example, because measures of segregation describing conditions at a regional level might not reflect people’s local experiences, a more useful measure might consider local conditions relative to the larger region, such as a LISA. However, co-ethnic exposure can exert effects on different geographic scales; residential communities can come together over block parties, assigned public school boundaries, and voting districts. Research in this area will benefit from studies that define residential communities using a variety of different scales, grounded in an explicit, theoretical model. Whatever measure of co-ethnic exposure is selected should be supplemented with measures to clarify a causal pathway, such as social capital (e.g., social support, social networks, neighborhood trust), feelings of belonging and connectedness, or built environment features (e.g., social or cultural institutions).

Additionally, existing measures do not capture the social forces that led to higher co-ethnic exposure. Co-ethnic exposure may be associated differently with CMH if it arose from historical, discriminatory forces or from individual decisions to live around neighbors of similar ethnic background. Similarly, associations may depend on other population characteristics – for example, country of origin and generational status [98]. Hence, research on neighborhood effects requires not only that “ethnic density” be disentangled from “immigrant concentration,” but also that they examine how heterogeneity within groups (e.g., immigration generation) may modify such effects.

#### Adjustment considerations

In studies of co-ethnic exposure, residual confounding is possible from other aspects of neighborhood deprivation or deficits in the built environment that affect health behaviors. Over-adjustment is also possible if co-ethnic exposure affects health behaviors or psychosocial factors that are included as model covariates. Thus, the selection of covariates to adjust for should be based on an explicit model. Socioeconomic disadvantage merits special consideration given its potential roles as both a cause and a consequence of high co-ethnic exposure, and its potential to either exacerbate or moderate the effects of co-ethnic exposure. An explicit causal model may necessitate presenting results both unadjusted and adjusted for socioeconomic disadvantage, and/or results for joint effects of socioeconomic disadvantage and co-ethnic exposure.

#### Causal inference

Most of the studies in this review were cross-sectional, unable to establish whether exposure to a neighborhood with a particular ethnic composition preceded the development of CMH outcomes, and thus unable to differentiate correlative and causal associations. This limitation is especially relevant for cardiometabolic conditions, which develop over many years and may reflect cumulative exposures across multiple residential environments – information not captured in studies that assess co-ethnic exposure and CMH outcomes at a single point in time. Residential self-selection is an alternative explanation, for example, and raises the problem of reverse causation – for example, if more physically or socially active individuals prefer neighborhoods with more resources, or if less active individuals are constrained to resource-poor neighborhoods. Longitudinal studies cannot fully eliminate the problem but, particularly if they incorporate repeated measures, can capture changes in residence and cumulative exposures, assess whether neighborhood characteristics affect subsequent health trajectories, and help establish the temporal sequence. Obtaining detailed residential histories and motivations for residential selection may also be informative.

Self-selecting into neighborhoods may also result in a form of confounding that standard strategies such as adjustment cannot address [4, 99, 100]. Propensity score methods offer an alternative approach [53, 54, 101, 102], as do natural experiments that take advantage of either rapid changes in residence (e.g., Moving to Opportunity study [103]) or neighborhood (e.g., gentrification).

### Implications

A logical next step is to discern the conditions in which co-ethnic exposure is beneficial for public health and when it is not. Two questions are of particular relevance. First, under what conditions can co-ethnic exposure facilitate the building of social capital and social connectedness in ways that promote resilience to stressors such as gentrification or economic change? Second, at what point do socioeconomic disadvantage and disenfranchisement overwhelm the potential benefits of co-ethnic exposure? Others have documented numerous examples of Black neighborhoods with strong co-ethnic social structures that were eroded or extinguished by racist acts [104, 105] or racially discriminatory measures [106, 107]. Environmental injustices continue to threaten beneficial structures built through higher co-ethnic exposure [108, 109].

Taken together, our findings suggest some public health and urban policy strategies: protecting community social structures by supporting community-based organizations and cultural centers; preventing displacement and environmental injustices by supporting affordable housing policies and equitable investments in infrastructure; building community control and collective efficacy through participatory planning processes or by supporting community land trusts. For example, policymakers are increasingly recognizing the value of co-designing programs and policies with community members [110]. The Centers for Medicare & Medicaid Services’ Access rule [111] calls for Medicaid program design and policy decisions to be informed by the lived experience of program beneficiaries [112]. The primary policy implication is to support conditions in which co-ethnic communities can collectively participate in decisions that generate health-promoting resources while reducing the structural disadvantages that undermine those resources. Although this review focused on co-ethnic exposure, many of these strategies may be relevant more broadly because the underlying mechanisms—social connectedness, collective efficacy, and community control—are not limited to neighborhoods defined by shared racial or ethnic identity. Nevertheless, given the importance of structural factors in shaping the opportunities of any given community, any efforts, interventions, or programs should consider the unique, historical processes that led to the development of that community. Initiatives will likely have the best chance for success if they recognize the historically rooted, structural constraints, as well as the available resources on which the community can build.

### Conclusion

Our review highlights substantial heterogeneity in the association of co-ethnic exposure with CMH, and it suggests that both positive and inverse associations are possible. In Black and Hispanic populations, co-ethnic exposure is more likely to be linked to poorer CMH outcomes, but across all three race/ethnic groups examined in this review, a substantial proportion of studies showed health benefits. These heterogeneous findings are consistent with a conceptual framework that highlights the interactions among structural (dis)advantage, community stressors, and neighborhood resources, with CMH implications mediated through a physiologic stress pathway. The evidence base would be elevated with more explicit theoretical frameworks that specify mediating pathways and testable hypotheses; more studies in Asian and Indigenous populations and populations from countries other than the US; study designs that examine the association over time; and explicit consideration of the historical circumstances that led to a given population’s high residential co-ethnic exposure. A clearer understanding of the association of co-ethnic exposure with CMH is critical especially given continued racial segregation [113]. Such understanding is key to identifying neighborhood conditions that promote health and wellbeing across communities.
