# Supplementary material for: A systematic review of co-ethnic exposure and cardiometabolic outcomes in racial and ethnic minoritized populations
Source: Public Health Rev. 2026 Sep 1;47:1609690. doi: 10.3389/phrs.2026.1609690 (PMC13574725; doi:10.3389/phrs.2026.1609690)
Supplement: Supplementary file 1 [file DataSheet1.docx]

Supplementary file. Databases and search strings used in literature search (California, United States, 2026).

| Database | Fields searched | Search string |
| --- | --- | --- |
| Web of Science | Topic (title, abstract, author keywords, KeyWords Plus) | (“ethnic enclave” OR “ethnic density” OR “residential segregation” OR “racial segregation” OR “neighborhood environment”) AND (“cardiovascular disease” or “diabetes” OR “obesity” OR “body mass index” OR “weight”) |
| PubMed | All fields | (“ethnic enclave” OR “ethnic density” OR “residential segregation” OR “racial segregation” OR “neighborhood environment”) AND (“cardiovascular disease” OR “diabetes” OR “obesity” OR “body mass index” OR “weight”) |
